# Prognostic Value of Tumor Size in Patients with Remnant Gastric Cancer: Is the Seventh UICC Stage Sufficient for Predicting Prognosis?

**DOI:** 10.1371/journal.pone.0115776

**Published:** 2014-12-30

**Authors:** Jun Lu, Chang-ming Huang, Chao-hui Zheng, Ping Li, Jian-wei Xie, Jia-bin Wang, Jian-xian Lin, Qi-yue Chen, Long-long Cao, Mi Lin

**Affiliations:** Department of Gastric Surgery, Fujian Medical University Union Hospital, Fuzhou City, China; Duke Cancer Institute, United States of America

## Abstract

**Background:**

The 7th UICC N stage may be unsuitable for remnant gastric cancer (RGC) because the original disease and previous operation usually cause abnormal lymphatic drainage. However, the prognostic significance of the current TNM staging system in RGC has not been studied.

**Methods:**

Prospective data from 153 RGC patients who underwent curative gastrectomy from Jan 1995 to Aug 2009 were reviewed. All patients were classified according to tumor size (<3 cm as N0;>3&≤5 cm as N1;>5&≤7 cm as N2; and>7 cm as N3). The overall survival was estimated using the Kaplan-Meier method, and hazard ratios (HRs) were calculated using the Cox proportional hazard model.

**Results:**

Tumor sizes ranged from 1.0 to 15.0 cm (median 5.0 cm). Tumor size, depth of invasion and lymph node (LN) metastasis were significant prognostic factors based on both the univariate and multivariate analyses (P<0.05). In the survival analysis, the seventh edition UICC-TNM classification provided a detailed classification; however, some subgroups of the UICC-TNM classification did not have significantly different survival rates. The combination of the seventh edition T classification and the suggested N classification, with ideal relative risk (RR) results and P value, was distinctive for subgrouping the survival rates except for the IA versus IB and II A versus IIB. A modified staging system based on tumor size, predicted survival more accurately than the conventional TNM staging system.

**Conclusions:**

In RGCs, tumor size is an independent prognostic factor and a modified TNM system based on tumor size accurately predicts survival.

## Background

Remnant gastric cancer (RGC) was originally defined as a gastric cancer detected more than 5 years after a distal gastrectomy for benign disease, and it was first described in 1922 [1]–[2]. Recently, in Eastern countries, this term has been used to define all cancers arising from the remnant stomach after partial gastrectomy, regardless of the initial disease or operation, and it includes local recurrence in the gastric stump after partial gastrectomy for gastric cancer [3]. Therefore, in the present study, RGC is defined as an adenocarcinoma of the stomach occurring 10 or more years after gastrectomy for benign disease or cancer [4]. As the time from initial gastric resection increases, the incidence of remnant cancer also increases [5]. The incidence of RGC ranges between 2.4% and 6% for all gastric cancer patients in Western centers [6], and it is 1–2% in Japan [7]. Due to its low incidence, there is limited prognostic information available to help guide the treatment of patients with RGC.

Lymph node (LN) metastasis is the most common metastatic pattern of RGC, and regional lymphadenectomy is recommended as part of radical gastrectomy[8], [9]. It was recently acknowledged that the total number of metastatic LNs is a more reliable prognostic indicator than positive anatomical lymphatic stations [10]. The N category, based on total number of metastatic LNs, and TNM staging are the most important prognostic factors in gastric cancer. Since 1997, the requirement of 15 or more dissected nodes for a pathological examination to accurately evaluate the status of the nodal metastasis and inhibit stage migration was proposed by the UICC and AJCC [11]. However, because of the initial partial gastrectomy removal of LNs, the total number of LNs and the perigastric LN metastasis rate were lower than for conventional gastric cancer, and it may be much more difficult to acquire 15 or more lymph nodes during operations for RGC [9], [12]. Most studies have focused on the prognosis of RGC based on the UICC/AJCC TNM system, and no previous studies have been conducted on the TNM stage itself. Hence, the suitability of the UICC N Stage of gastric cancer for predicting the overall survival of RGC had to be reconsidered.

In patients with lung, breast or thyroid cancer, tumor size is one of the major components of the TNM cancer staging scheme, which is in addition to lymph node metastasis and distant metastasis [13]. However, the prognostic value of tumor size in patients with gastric cancer remains controversial [14]. Recently, some authors [15], [16] have demonstrated that tumor size is an independent prognostic indicator in gastric cancer, and tumor size is a simple and practical prognostic factor in patients with gastric cancer. Our previous study suggested that tumor size might supplement clinical staging in the future [17].

In this study, we retrospectively analyzed the tumor sizes of RGC patients who underwent curative gastrectomies and evaluated the prognostic significance of tumor size. The other main aim of the present study was to evaluate survival differences between the subgroups in the current staging system; we developed a modified TNM system based on the tumor size as well and, compared the survival curves between the two systems (seventh UICC system vs the modified system).

## Methods and Materials

The ethics committee of Fujian Medical University Union Hospital approved this retrospective study. Patient records/information were anonymized and de-identified prior to the analysis. Written informed consent was provided by participants (or next of kin/caregiver in the case of children) for their clinical records to be used in this study.

RGC was defined as a carcinoma of the stomach occurring 10 or more years after distal gastrectomy for benign disease or cancer [4], [18]. A minimal latency of 10 years was chosen to avoid spurious effects due to faulty diagnosis of recurrent cancers and latent carcinoma, that were not detected in the initial operation [19].

Between Jan 1995 and Aug 2009, 3021 patients with gastric carcinoma were treated at the Department of Gastric Surgery, Fujian Medical University Union Hospital. During that period, 172 patients (5.7%) underwent surgical resection for RGC. Among them, 19 patients with insufficient clinical and/or histopathologic data, double primary cancers, distant metastasis, proximal (non-distal) gastrectomy or non-curative resection for initial diseases were excluded. The medical records of 153 patients were reviewed for the following information: the demographic factors, diagnosis of initial disease, reconstruction of the first operation, follow-up method, characteristics of the RGC (histology, gross type, harvested LNs, tumor size and stage), and follow-up data. The clinical, pathological, and surgical findings for the RGC patients were collected retrospectively from our prospectively acquired database.

According to the initial gastric diseases in each patient, RGC was classified as either a RGC after a distal gastrectomy for benign disease (RGC-B) or as RGC following gastric cancer (RGC-C). The histology was categorized as differentiated (papillary, well differentiated, and moderately differentiated carcinoma) or undifferentiated (poorly differentiated, mucinous adenocarcinoma, and signet ring cell carcinoma) [3]. The gross type was recorded in accordance with the Japanese Classification of Gastric Carcinoma [20]. TNM classification was applied according to guidelines from the International Union Against Cancer (UICC) (7th Edition, 2010) [21]. Tumor size was measured according to the Japanese Classification of Gastric Carcinoma [20], and the longest tumor diameter was measured and used in this study as we reported in a previous study [17].

### Follow-up and survival analysis

Postoperatively, patients were examined at follow-up visits every 3 months for the first 2 years and every 6 months thereafter. At each follow-up, the carcinoembryonic antigen (CEA) and carbohydrate antigen 19-9 (CA199) levels were determined. Thoracicoabdominal and pelvic computed tomographic scan or abdominal ultrasonography was performed every 3–6 months. Gastroscopy was performed annually. All surviving patients were followed for more than five years. The overall survival (OS), defined as the time from operation to death or final follow-up, was used as a measure of prognosis. The median follow-up period of the 153 patients was 47.2 months, ranging from 2 to 186 months.

For the statistical analysis, the Chi-square tests were used for categorical variables. Survival curves were estimated using the Kaplan-Meier method and were compared with the log-rank test. All of the statistically significant variables observed in the univariate analysis were included in the multivariate survival analysis using the Cox proportional hazard model. Relative risk (RR) is the ratio of the risk of death from cancer in the group exposed to the factor to that in the unexposed group. We calculated the RR with the Cox proportional hazards model in SPSS survival analysis using the forward logistic regression stepwise procedure. Predictive accuracy estimates were then compared between the UICC-TNM stage and modified TNM stage model, which include tumor size. The hazard ratio and its 95% confidence interval (CI) were assessed for each factor. A value of P<0.050 (two-sided) was considered statistically significant. Statistical analysis was performed using SPSS version 17.0 (SPSS Inc., Chicago, IL).

## Results

### Clinicopathological Features of Remnant Gastric Cancer

The detailed characteristics of the 153 patients are listed in Table 1, the cohort consisted of 111 (72.5%) males and 42 (27.5%) females; the median age was 61 years (range 41–80 years). Their primary diseases were gastric cancer (121; 79.1%) and benign disease (32; 20.9%). The type of the reconstruction method of first gastrectomy was Billroth I (108; 70.6%) and Billroth II (45; 29.4%). According to the histology of RGCs, 114 (74.5%) cases were differentiated and 39 (25.5%) were undifferentiated. The invasion depth of 153 patients was pT1 in 16 (10.4%) patients, pT2 in 30 (19.6%) patients, pT3 in 49 (32.0%) patients, and pT4 in 58 (37.9%) patients. Sixty-seven (43.7%), 31 (20.3%), 42 (27.5%), and 13 (8.5%) patients had N Stages of N0, N1, N2, and N3, respectively. In this study, 7 (4.6%) tumors were Borrmann type I, 45 (29.4%) Borrmann type II, 84 (54.9%) Borrmann type III and 17 (11.1%) Borrmann type IV.

**Table 1:** Univariate analysis of 5-years survival rate.

Variable	n	5-years survivalrate (%)	P value
Age (years)			0.203
<60	60	40.0	
≥60	93	37.7	
Sex			0.222
Male	111	36.9	
Female	42	28.6	
Depth of invasion			<0.001
T1	16	87.5	
T2	30	57.3	
T3	49	36.7	
T4	58	6.9	
Lymph node stage			<0.001
N0	67	44.8	
N1	31	35.5	
N2	42	25.6	
N3	13	7.7	
Histological types			0.562
Differentiated	114	35.9	
Undifferentiated	39	30.8	
Borrmann type			0.195
I	7	57.1	
II	45	37.8	
III	84	33.1	
IV	17	23.5	
Number of LNs obtained			0.064
<15	59	28.8	
≥15	94	38.2	
Initial disease			0.105
Gastric cancer	121	33.8	
Benign lesion	32	37.5	
Reconstruction of first operation			0.183
B-I	108	37.0	
B-II	45	28.9	
Tumor size(cm)			<0.001
≤3	43	65.1	
>3&≤5	42	38.1	
>5&≤7	36	16.7	
>7	32	12.5	

### Tumor size

The tumor size ranged between 1.0 and 15.0 cm (mean 5.4 cm and median 5.0 cm). The tumor size was then classified into quartiles as ≤3 cm,>3 & ≤5 cm,>5 & ≤7 cm, and >7 cm.

### Univariate Analysis

The 5-year overall survival (OS) rate was 34.6% for all 153 patients. In addition to the tumor size, the significant prognostic factors included the depth of invasion and lymph node status. Table 1 showed findings from the univariate analysis for prognostic factors.

### Multivariate Analysis

Multivariate survival analysis, including all statistically significant prognostic factors mentioned in univariate analysis, was performed to determine the independent prognostic factors for RGC. Multivariate analysis with the Cox proportional hazard model showed that the tumor size was an independent prognostic factor as were the depth of invasion and lymph node status (Table 2).

**Table 2:** Multivariate analysis with 5-years survival in RGC patients.

Variable	HR	95%CI	P value
Lymph node stage			0.007
N0	1	Reference	
N1	1.115	0.613–2.027	0.722
N2	1.918	1.077–3417	0.027
N3	3.360	1.580–7.146	0.002
Depth of invasion			<0.001
T1	1	Reference	
T2	2.623	0.571–12.059	0.215
T3	3.465	1.135–18.968	0.048
T4	10.326	2.393–44.554	0.002
Tumor size(cm)			<0.001
≤3	1	Reference	
>3&≤5	1.125	0.553–2.286	0.745
>5&≤7	3.133	1.604–6.120	0.001
>7	6.749	3.473–13.115	<0.001

### Comparison of Survival According to the UICC-TNM Stage and Modified TNM Stage

**Figure 1:**
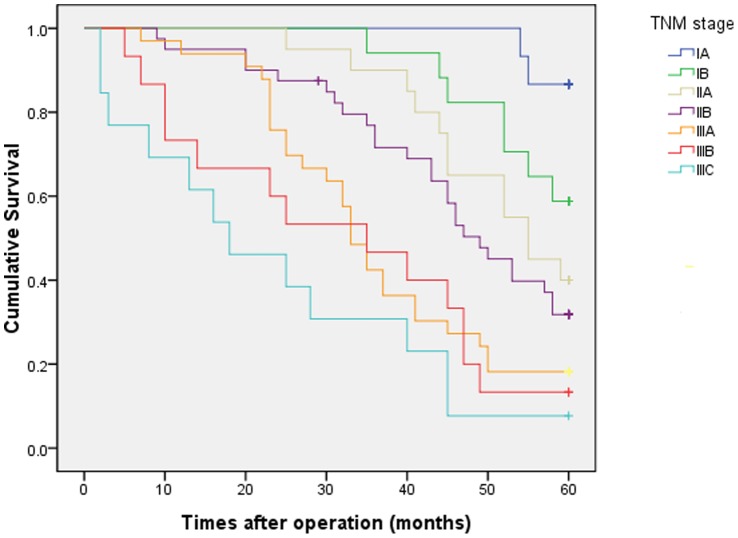
Kaplan-Meier survival curves of RGC stratified by the 7th UICC staging system.

**Figure 2:**
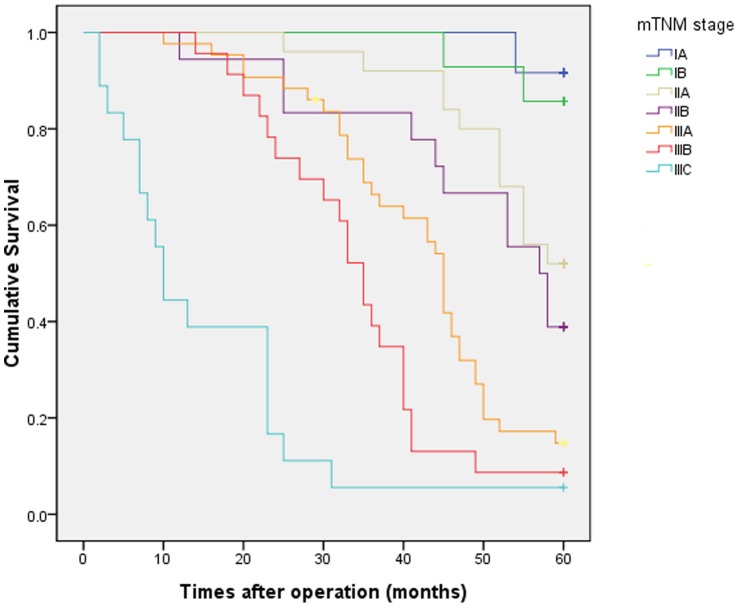
Kaplan-Meier survival curves of RGC with modified staging based on tumor size (≤3 cm as N0; >3&≤5 cm as N1; >5&≤7 cm as N2; and >7 cm as N3) instead of current lymph node stage(N stage).

According to the 7th UICC-TNM stage, the 5-year OS rate of patients at stages IA, IB, IIA, IIB, IIIA, IIIB, and IIIC were 86.7%, 58.8%, 40.0%, 31.8%, 18.2%, 13.3%, and 7.7%, respectively (Fig. 1). However, the number of cases with fewer than 15 removed LNs was high (59, 38.6%), and the number of cases with more than 7 metastatic LNs was low (13, 8.5%) in this study. Therefore, it may be unreasonable to use a cut off of 15 total LNs and 7 metastatic LNs as required by UICC-TNM. We then constructed a modified TNM stage (mTNM stage) based on the tumor size (<3 m as N0;>3&≤5 cm as N1;>5&≤7 cm as N2;and >7 cm as N3) instead of the current lymph node stage (UICC N stage). Patients with stages IA, IB, IIA, IIB, IIIA, IIIB, and IIIC mTNM presented with 5-year OS rates of 91.7%, 85.7%, 52.0%, 38.9%, 14.8%, 8.7%, and 5.6%, respectively (Fig. 2). The all subgroups of the seventh edition TNM staging system based on metastatic LNs did not distinguish between significantly different survival rates, except for stage IIB versus IIIA (P  = 0.027). However, the cumulative survival curves according to the modified stage were well separated, except for IA versus IB and IIA versus IIB (P  = 0.648 and P  = 0.369, respectively). We then demonstrated the more appropriate N stage based on tumor size (Table 3).

**Table 3:** Detailed postoperative survival differences between the 7th UICC TNM stage and our suggested category of the tumor size.

The 7th UICC TNM stage				Modified TNM stage based on tumor size			
TNM	OS (%)	RR	P value*	mTNM	OS (%)	RR	P value*
IA	86.7	1	–	IA	91.7	1	–
IB	58.8	3.773	0.098	IB	85.7	1.750	0.648
IIA	40.0	1.748	0.241	IIA	52.0	4.042	0.042
IIB	31.8	1.364	0.375	IIB	38.9	1.455	0.369
IIIA	18.2	1.875	0.027	IIIA	14.8	2.179	0.024
IIIB	13.3	1.180	0.626	IIIB	8.7	1.937	0.019
IIIC	7.7	1.564	0.271	IIIC	5.6	3.208	0.001

*Comparison of the OS with the former TNM stage.

### Predicting the 5-year Overall Survival

The accuracy of the UICC 7th TNM staging system in predicting the 5-year OS rate was 73.9%, whereas the modified TNM stage based on tumor size increased the accuracy of predicting the 5-year OS rate to 77.8%, and the modified staging system more accurately predicted survival (Table 4).

**Table 4:** Multivariate regression models predicting 5-year overall survival, according to 7th UICC and modified TNM stage classification.

Variable	5-years survival					
	7th TNM classification			modified TNM classification		
	OR	95%CI	P value	OR	95%CI	P value
II stage vs I stage	4.414	1.737–11.216	0.002	8.817	2.299–33.809	0.002
III stage vs I stage	14.765	5.817–42.035	<0.001	50.087	10.709–123.838	<0.001
Predictive accuracy of the model (95%CI)	73.9% (66.9%–80.9%)			77.8%(71.2%–84.4%)		
Increased predictive accuracy (95%CI)	–			+3.9%(0.8%–7.0%)		

## Discussion

RGCs are often detected at advanced stages and have poor prognosis with 5-year survival rates ranging from 40% to 60% [22], [23]. Based on our experience, the overall 5-year survival rate was 34.6% and seems to be worse than in recent Western series [6], [24], but this result might be related to the relatively low rate of early stage disease (10.4%) compared to Di et al [6], who reported that the rate of early stage disease was 25% and the 5-year survival rate was also approximately 35% for patients with advanced forms. A study by Thorban et al [25] also supported the finding that RGC patients with UICC stage IA disease have a considerably better prognosis than patients with advanced tumors. Consequently, the performance of lifelong annual follow-up endoscopic examinations after the initial gastrectomy should be emphasized, and endoscopic diagnosis of early lesions may offer the best hope for cure.

Metastatic LNs are a well-established prognostic factor for gastric carcinoma [25]. Although we are unable to study the pattern of lymphatic tumor spread in this retrospective study, others have investigated this phenomenon [5], [26]. Many researchers have suggested that the RGCs and upper third primary gastric tumors have different lymphatic spread [6], [27], [28]. As Di et al [29] reported in a previous study, the main lymphatic flow drains from a tumor located in the upper third of the stomach into nodes along the lesser curvature, the right cardia, the left gastric artery, and the celiac artery. However, in RGC, these lymphatic pathways have been cut off. Previous partial gastrectomy usually causes lymphatic leakage, blockage, and regeneration of lymphatic flow around the gastric stump as well as induces abnormal lymphatic formation [30]. Indeed, complete removal of the remnant stomach plus D2 lymphadenectomy is still the optimal procedure. However, formal, adequate lymphadenectomy in patients with RGC for staging (at least 15 lymph nodes) may be more difficult because of prior gastric resection [5].

Ideal cancer staging should not only provide an indication of the prognosis and a framework for treatment decisions, it should also allow for evaluation of treatment with meaningful comparisons between different treatments or the same treatment modalities according to different groups[31]. The tumor-node metastasis (TNM) staging system, which incorporates the tumor depth, nodal involvement, and metastatic status for solid tumors and cancer including RGC staging [3], [5], [7], is widely accepted. Since 2010, the 14th edition of the Japanese Gastric Cancer Association (JGCA) staging system officially released an abandoned anatomic nodal classification and adopted numeric classification identical to the UICC/AJCC TNM system; when utilizing the current UICC N staging system, more than 15 retrieved lymph nodes are required for optimal staging [20]. In many studies, the 7th UICC N staging system has been superior to the 5th/6th UICC N stage and Japanese N stage for prognostic prediction of gastric cancer using Cox regression multivariate analysis [32]. However, because of initial distal gastrectomy and the removal of LNs, the total number of LNs and the level of perigastric metastatic LNs was lower than conventional gastric cancer[5], [9], [27]. These findings are also demonstrated in the study by Rabin et al [12]. Our results were consistent with the results of previous studies. The explanation is most likely based on the fact that a substantial number of nodes had been harvested during the primary resection.

Some authors have noted that stage migration may be an issue with TNM staging systems [33], [34]. If the number or level of the retrieved lymph nodes is insufficient, stage migration is observed in 10% to 15% of cases [35]. On the other hand, the number of metastatic lymph nodes (MLNs) may be underestimated if only a few lymph nodes are removed [36]. In our small sample study, the number of MLNs was ≥7 in a few patients (13/153,8.5%), and the total number of harvested LNs was ≥15 in some patients (94/153,61.4%). We examined the prognostic stratification according to the seventh UICC/AJCC Cancer Staging Manual within each stage. However, unexpectedly, the cumulative survival curves according to each of the seventh edition TNM stages were insufficiently separated. We failed to demonstrate a significant difference in the 5-year survival rate between each subgroup except for IIB and IIIA.

Among several clinicopathologic factors, the tumor size can easily be measured before or during the operation without requiring special tools [37]. In a Japanese study [37], the tumor size was strongly correlated with the parameters of tumor progression, such as the depth of invasion, degree of lymph node metastasis, and stage of the disease. Wang et al [38] suggested that tumor size could efficiently and reliably reflect the lymph node status. We previously demonstrated that [16] tumor size is a predictor of preoperative N staging in T2-T4a stage advanced gastric cancer. Saito et al [39] reported that tumor size might be a good indicator in the prediction of recurrence site as well as serve as a simple predictor of survival of patients with gastric cancer. In this study, multivariate analysis revealed that tumor size independently influenced patient survival. These results indicate that tumor size provides important information about the malignant potential of tumors.

Interestingly, in this study, the Cox multivariate analysis showed that the novel N classification based on tumor size was superior to the seventh edition N classification as an independent prognostic factor. Therefore, we presumed that the 7th UICC N stage might be an unsuitable prognostic factor and that it should be evaluated and improved to help surgeons rationally estimate the TNM stage. From the prognostic analysis of current staging systems for gastric cancer, we first proposed a novel staging system that was combined with the seventh edition T and the M classification and the suggested N classification based on tumor size. All classes in the suggested final classification were associated with significant differences in the cumulative survival rates except for IA and IB as well as IIA and IIB, between which there was no significant difference. From this perspective, the novel staging system demonstrated better discrimination than the current UICC TNM classification. Furthermore, in the present study, the suggested TNM staging system increased the prognostic predictive accuracy by 3.9% with a 95% CI of 0.8–7.0%.

An accurate cancer staging system is crucial in clinical practice. It can help clinicians as they select treatment plans and compare treatment results among institutions and countries [40]. Although our sample size was small, we found that a novel TNM classification, composed of the seventh edition T classification and modified N classification based on tumor size, may provide a better stratification of prognosis than the current systems for RGC patients. From a clinical standpoint, the current results are important and may improve the prognostic power of the current TNM staging system, ultimately refining the selection of patients who may benefit the most from adjuvant treatments.

The limitations of this study include its retrospective design and the fact that we included only few RGC revisions from a single institution. Future large-scale studies are required to validate our findings. However, the proposed TNM stage system offers a simple and reliable method to stratify RGC patient survival in stages II and III, and it does not require any special techniques or biomarkers.
